# Unexpected decrease in necrotizing enterocolitis morbidity during the COVID-19 pandemic-A single-centre retrospective study

**DOI:** 10.3389/fped.2024.1346478

**Published:** 2024-05-28

**Authors:** Yu Wang, Mingling Cui, Lili Li, Chuchu Gao, Zongtai Feng, Yan Cai, Zuming Yang, Lirong Shen

**Affiliations:** Department of Neonatology, The Affiliated Suzhou Hospital of Nanjing Medical University, Suzhou, Jiangsu, China

**Keywords:** COVID-19, necrotizing enterocolitis (NEC), morbidity, pathogen, antimicrobial susceptibility

## Abstract

**Background:**

The impact of the coronavirus disease 2019 (COVID-19) pandemic on neonatal necrotizing enterocolitis (NEC) is not well characterised. This cross-sectional study evaluated the potential effects of pandemic-related measures on NEC morbidity in premature infants in a neonatal ward during the COVID-19 pandemic.

**Methods:**

This was a retrospective study conducted in a tertiary neonatal ward in eastern and central China over 6 consecutive years (2017, 2018, 2019, 2020, 2021 and 2022). The medical records of 189 premature infants with stage II or III NEC were reviewed for clinical manifestations and aetiologies. The data were analysed and compared between the prepandemic period (2017, 2018, and 2019) and the pandemic period (2020, 2021 and 2022).

**Results:**

A total of 9,903 infants with gestational age (GA) < 37 weeks were enrolled, including 5,382 in the prepandemic period and 4,521 in the pandemic period. A reduction in stage II or III NEC morbidity was observed in infants with GA < 37 weeks, with an average annual morbidity of 2.29% (123/5,382) (95% CI, 1.89%–2.68%) in the prepandemic period and 1.46% (66/4,521) (95% CI, 1.11%–1.81%) in the pandemic period. NEC morbidity showed resurgent characteristics in 2021. When prepandemic coinfections were excluded, most cases of NEC with bloodstream infections in the prepandemic period were attributable to Gram-negative bacteria (27/32, 84.38%), mainly *Klebsiella pneumoniae*, while in the pandemic period they were attributable to Gram-positive bacteria (10/18, 55.56%), mainly *Staphylococcus aureus*. Antimicrobial susceptibility testing revealed that *Klebsiella pneumoniae* was 100% sensitive to meropenem, imipenem, ciprofloxacin and levofloxacin and 100% resistant to ampicillin. *Staphylococcus capitis* was 100% sensitive to vancomycin, linezolid, tetracycline, cotrimoxazole and cefoxitin and 100% resistant to penicillin and benzathine.

**Conclusions:**

COVID-19 pandemic-related interventions can reduce the morbidity of NEC and change the pathogen spectrum in patients with bloodstream infections. We need to understand the exact factors leading to these changes.

## Introduction

Cases of novel coronavirus infection were first reported in December 2019 in Wuhan, Hubei province, China (1). In late January 2020, the Suzhou Municipal Government implemented the first-level public health emergency response and ordered a stringent lockdown. More rigid infection prevention strategies, such as visitor restrictions, environmental disinfection, hand hygiene, and the use of personal protective equipment, were implemented in neonatal wards. An ecological analysis (2) comparing the incidence of 31 major notifiable infectious diseases in 2020 to the average level during 2014–2019 showed that strong nationwide non-pharmaceutical interventions were associated with a broad diminution effect on communicable diseases. Kenji Hirae (3) investigated the epidemiology of 36 communicable diseases and found that the number of cases of the 27 diseases caused mainly by contact- or droplet-transmitted pathogens was lower in the pandemic period (2020–2021) than in the prepandemic period (2015–2019), and the number of cases of 21 diseases continued to decrease in the pandemic period.

The financial impact of necrotizing enterocolitis (NEC) is estimated to be $1 billion per year in the US alone. NEC and surgery for NEC are associated with an increased risk of adverse neurodevelopmental outcomes at two years of age (4). The development of the infant microbiome is highly dynamic and easily influenced by maternal, host, and environmental factors (5). Numerous studies have shown an association between NEC and the intestinal microbiota (6). The presence of Epstein‒Barr virus and adenovirus may be related to the severity of NEC (7). The association between human coronavirus infection and neonatal NEC has also been reported in several studies (8–10). However, whether pandemic-related measures have clinical implications for major neonatal morbidities, such as NEC morbidity, in preterm infants has not yet been studied. In this study, we hypothesised that: (1) during the three-year pandemic period when non-pharmaceutical interventions were implemented, the average annual incidence of NEC would decrease; and (2) pathogens would be affected.

The Suzhou Municipal Government of China enacted lockdown measures at the end of January 2020 and relaxed these non-pharmaceutical interventions on 10 December 2022. In this study, we took 31 January 2020, as the time node and compared the morbidity and aetiology of NEC over nearly six years, i.e., from 22 March 2017, to 31 January 2020 (the prepandemic period), and from 1 February 2020, to 10 December 2022 (the pandemic period).

## Research subjects and methods

### Study site

The Affiliated Suzhou Hospital of Nanjing Medical University is a large, 110-bed, maternal and child health and free-standing tertiary referral centre in an urban setting in Suzhou, China, with over 20,000 newborn deliveries per year. Suzhou is an important central city in the Yangtze River Delta, and its level of economic development is higher than the average for all cities in China.

### Patients and samples

We conducted a retrospective analysis of data from 9,903 premature infants who were admitted to the Affiliated Suzhou Hospital of Nanjing Medical University with a final diagnosis of stage II and III NEC during the period between 22 March 2017 and 10 December 2022. The demographic and clinical data of preterm infants born in 2017 and 2022 with stage II and III NEC were collected to compare the incidence and aetiology of NEC. The mean GA at diagnosis of NEC was 33.46 weeks (SD 2.52) corrected gestational age. The mean NEC onset and NEC diagnosis times were 17.8 ± 0.92 d and 18.7 ± 0.91 d, respectively. Overall, 54.0% of the newborns were male. Approximately 11.6% (22/189) of the deliveries were artificial insemination, 54.5% (103/189) were by caesarean section, and 25.9% (49/189) were multiple gestations. The inclusion criteria for this study were as follows: (1) infants aged <28 days, (2) infants with a gestational age (GA) <37 weeks, and (3) infants who met the criteria for stage II and III NEC. NEC was diagnosed according to Bell's diagnostic criteria (11). In preterm infants who presented with NEC before first breastfeeding, intrauterine factors were considered first by clinicians. This happened before the pandemic-related measures in the NICU. We therefore excluded this group of infants. We also excluded preterm infants with spontaneous intestinal perforation (SIP), which is similar to NEC, but the pathophysiology of SIP is different from NEC and typically presents as focal intestinal perforation at the terminal ileum (12).

### Reagents and microbiological sampling

Bacterial isolates were conﬁrmed by classical microbiological methods including catalase and Gram stain. Species identification and initial drug susceptibility were further identiﬁed by biochemical characterisation using the VITEK 2 Compact test (bioMérieux, Lyon, France). Staphylococcus aureus ATCC 29,223 and Escherichia coli ATCC25922 were served as quality control strains. Meropenem, imipenem, ceftriaxone, ampicillin, piperacillin/tazobactam, gentamicin, quinolones, linezolid and vancomycin were purchased from Oxoid Ltd (Basingstoke, United Kingdom). The minimum inhibitory concentrations of the antibiotics were performed by the broth dilution method, the E test (bioMérieux), or the disk diffusion method according to the Clinical and Laboratory Standards Institute guidelines. In our laboratory, the broth dilution method is mainly used for antimicrobial susceptibility testing. Using a sterile loop, three to five colonies of the isolate under investigation were picked from pure culture and suspended in three mL of demineralised water. The turbidity of the suspension was standardised to a 0.5 McFarland standard. Then 50 µl of the bacterial suspension was transferred to 11 ml of Mueller-Hinton broth using a micropipette. The tubes containing the broths were then mixed using a vortex mixer. Using an automated inoculation delivery system (Thermo Scientific Sensititre AIM Automated Inoculation Delivery System), 100 ul of broth was dispensed into each well of the microplate. The microplates were covered with an adhesive film and incubated at 37 °C for 18–24 h. The MICs of each antimicrobial molecule were determined using a semi-automated system, the Vizion (Thermo Scientific Sensititre Vizion Digital MIC Viewing System), which provides digital images of the plate (13).

### Definition of NEC

The diagnosis of NEC was based on the documentation of the attending neonatologists who cared for the infant at the time. Two of the authors (YW and MLC) reviewed the documentation when collecting the clinical material according to the medical records and reviewed the final diagnosis after following up those who were then transferred to the surgical ward. Infants who met the following criteria were included in the NEC group: (1) had one or more systemic symptoms, including an unstable body temperature, bradycardia, drowsiness and apnoea; (2) had one or more clinical signs, including gastric aspirate with bile or emesis, abdominal distention, and occult and/or obvious blood in the stool; and (3) at least one imaging finding, including portal vein gas, pneumatosis intestinalis, and/or pneumoperitoneum. Infants were classified as having stage I, II, or III severe NEC according to Bell's classification standard (14, 15). Infants with stage II and III NEC were included in this study.

### Quality control and ethics

Data were collected from the electronic medical record system by one author and checked by the other author. Two of the authors reviewed the charts. The infant/nurse ratio remained essentially the same over the years, and we did not change the type of hand sanitizer. The study was conducted in conformity with the principles and regulations of the Helsinki Declaration and was approved and reviewed by the Medical Ethics Committee of the Affiliated Suzhou Hospital of Nanjing Medical University (approval number of ethical documents: K-2020-086-K01).

### Statistical analysis

The chi-square test, two independent sample T tests and Mann‒Whitney test were performed in the Statistical Package for the Social Science (version 22.0, IBM Corp, Armonk, NY, USA) to compare the demographic characteristics between the 3-year periods (2017–2019 and 2020–2022). Line graphs were used to describe the annual incidence rates of stage II or III NEC in preterm infants before and during the COVID-19 pandemic. Differences with a *p* value of less than 0.05 were considered statistically significant.

## Results

### Study population and baseline data

**Figure 1:**
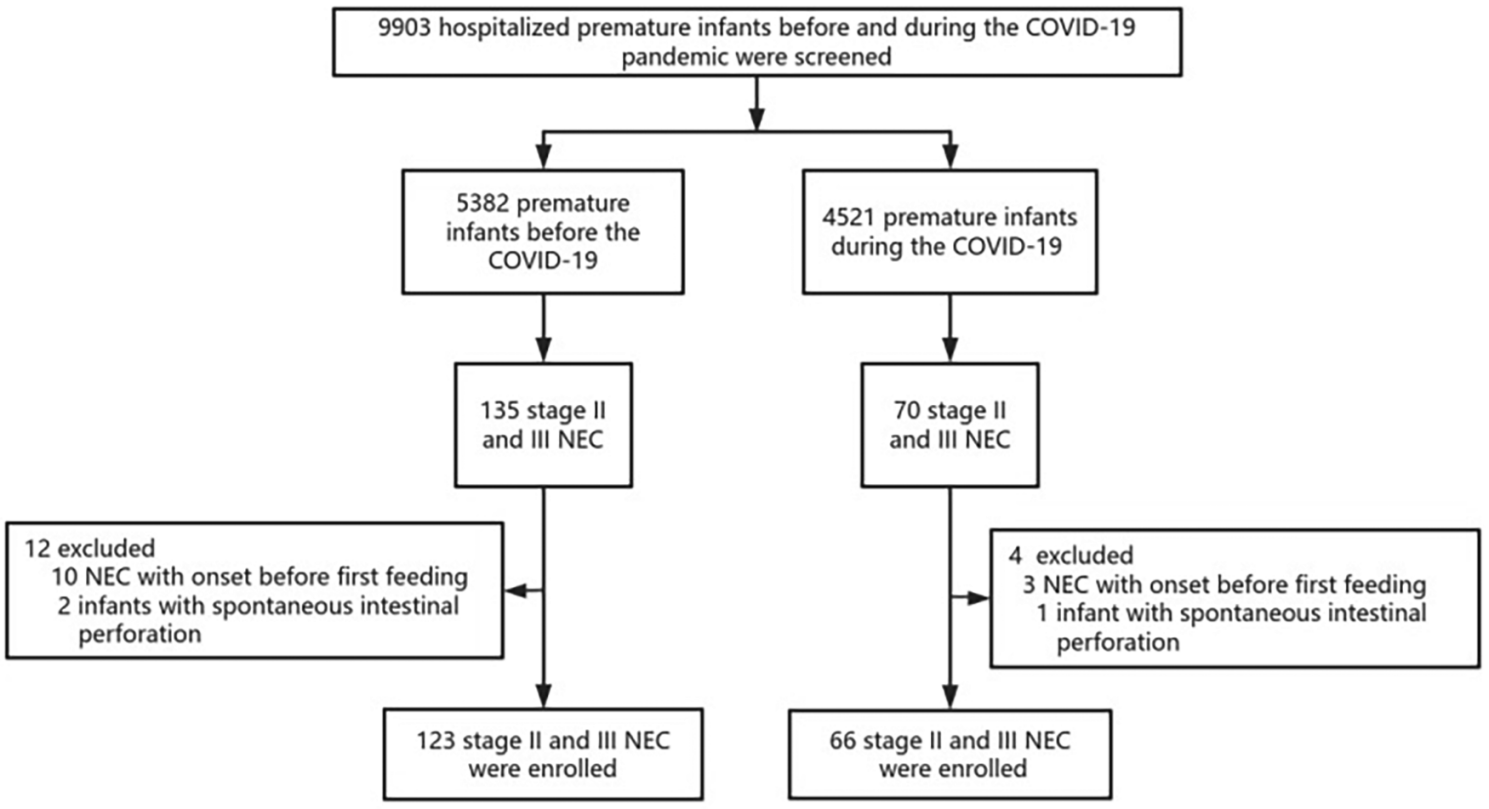
Flowchart of the study. A total of 189 infants with stage II or III NEC were included from 9,903 premature infants with GA < 37 weeks in the study. NEC, necrotizing enterocolitis; COVID, coronavirus disease.

Data from the 9,903 premature infants with GA < 37 weeks were reviewed. A total of 189 premature infants with stage II or III NEC were finally included in the study: 123 born in the prepandemic period and 66 born in the pandemic period. A flowchart depicting the study process is shown in Figure 1. Included infants had a mean GA of 30.77 weeks (SD 2.84) and a mean birth weight (BW) of 1,300 (1,100, 1,650) g. None of the mothers tested positive for COVID-19 during pregnancy. Their demographic data are summarised in Table 1. No significant differences were noted in the baseline parameters between the two groups.

**Table 1:** Demographic characteristics in preterms with NEC between the two groups.

Variables	Prepandemic period	Pandemic period	*χ*^2^/t/Z	*P* value
GA, weeks	30.9 ± 2.80	30.5 ± 2.70	0.950^b^	0.343
Birth weight, g	1,350 (1100,1700)	1,300 (1087,1500)	−0.902^c^	0.367
Male	63 (51.22%)	39 (59.09%)	1.070^d^	0.301
Multiple gestation	32 (26.02%)	17 (25.76%)	0.001^d^	0.969
In vitro fertilisation	14 (11.38%)	8 (12.12%)	0.023^d^	0.880
HDP	30 (24.39%)	13 (19.70%)	0.538^d^	0.463
GDM	25 (20.33%)	15 (22.73%)	0.149^d^	0.700
Rupture of membranes >18 h	33 (26.82%)	16 (24.24%)	0.891^d^	0.342
Vaginal delivery	57 (46.34%)	29 (43.94%)	0.100^d^	0.752
Amniotic fluid contamination	8 (6.50%)	4 (6.06%)	0.000^a^	1.000
Small for gestational age	20 (16.26%)	14 (21.21%)	0.714^d^	0.398
1-min Apgar score	8.00 (6.00,9.00)	8.00 (7.00,9.00)	−0.996^c^	0.319
5-min Apgar score	9.00 (9.00,10.00)	9.00 (8.00,9.25)	−1.584^c^	0.113
PH of blood gas analysis on admission	7.27 (7.23,7.33)	7.28 (7.22,7.33)	−0.304^c^	0.761
BE of blood gas analysis on admission, mmol/L	−4.36 ± 3.83	−3.62 ± 4.35	−1.027^b^	0.229
Speed of feed, ml/kg/d	14.11 ± 5.58	13.79 ± 6.00	0.366^b^	0.715
Feeding				
Breast milk	25 (20.33%)	0 (0.00%)	20.221^d^	<0.001
Milk	81 (65.85%)	62 (93.94%)		
Breast milk and formula	17 (13.82%)	4 (6.06%)		
Feeding start time, day	2.00 (2.00,3.00)	2.00 (1.75,4.00)	−1.290^c^	0.197
NEC onset time, day	16.57 ± 12.30	20.15 ± 13.97	−1.820^b^	0.070
NEC diagnosis time, day	17.62 ± 11.39	21.02 ± 14.24	−1.744^b^	0.075
Ibuprofen	14 (11.38%)	4 (6.06%)	1.412^d^	0.235
Blood products transfusion within 72 h before onset	13 (10.57%)	10 (15.15%)	0.844^d^	0.358
Stage II	94 (76.43%)	52 (78.79%)	0.137^d^	0.712
Gram-positive	5 (15.63%)	10 (55.56%)	8.747^d^	0.003
Antibiotic exposure	62 (50.40%)	26 (39.4%)	2.094^d^	0.148
Central line associated blood stream infections	2 (1.60%)	0 (.00%)	–^e^	0.543
Late on set sepsis	81 (65.80%)	43 (65.20%)	0.009^d^	0.923

Data are expressed as the mean ± SD, median (interquartile range), or *n* (%). No significant differences were noted in the baseline parameters between the two groups. COVID, coronavirus disease; NEC, necrotizing enterocolitis; GA, gestational age; HDP, hypertensive disorders in pregnancy; GDM, gestational diabetes mellitus; BE, base excess.

^a^ Continuity correction.

^b^ Student's *t* test.

^c^ Mann–Whitney test.

^d^ Chi-square test.

^e^ Fisher's exact test.

### Incidence of NEC

There was an overall decreasing trend in NEC morbidity over the 6 years of this study. A reduction in NEC (stage II or III) morbidity in all premature infants was observed, with a reduction of 2.29% (123/5,382) (95% CI, 1.89%–2.68%) in the prepandemic period vs. 1.46% (66/4,521) (95% CI, 1.11%–1.81%) in the pandemic period, and this decline was statistically significant (*χ*^2 ^= 8.945, *P* = 0.003). No significant differences were noted in the constitutive rate of NEC between the two groups (*χ*^2 ^= 0.137, *P* = 0.712).

### Annual trends in NEC incidence from 2017 to 2022

**Figure 2:**
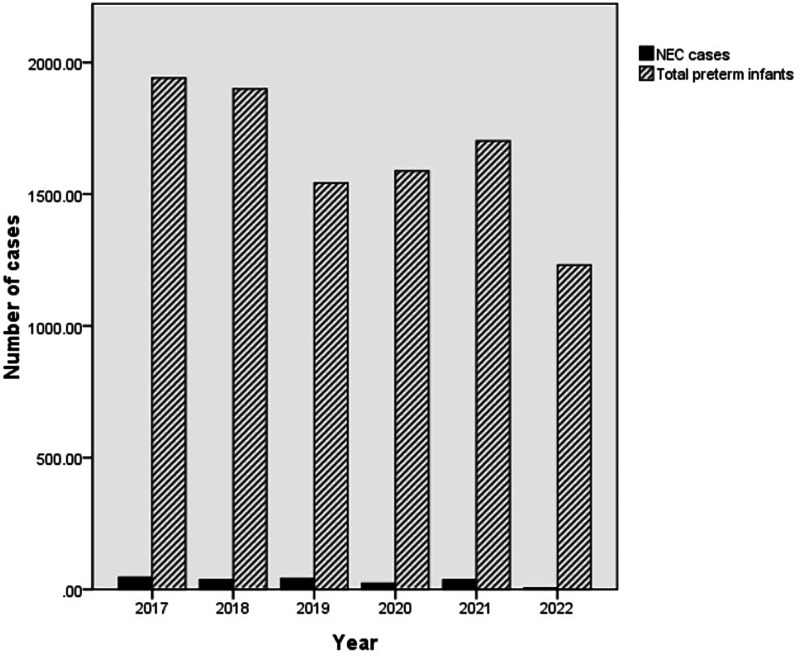
The total number of preterm infants and the number of *NEC* cases. The number of *NEC* patients (cases)/total number of preterm infants per year (cases) was 45/1,941 (2017), 37/1,899 (2018), 41/1,542 (2019), 23/1,588 (2020), 37/1,702 (2021) and 6/1,231 (2022) over the 6 years, respectively. NEC, necrotizing enterocolitis.

**Figure 3:**
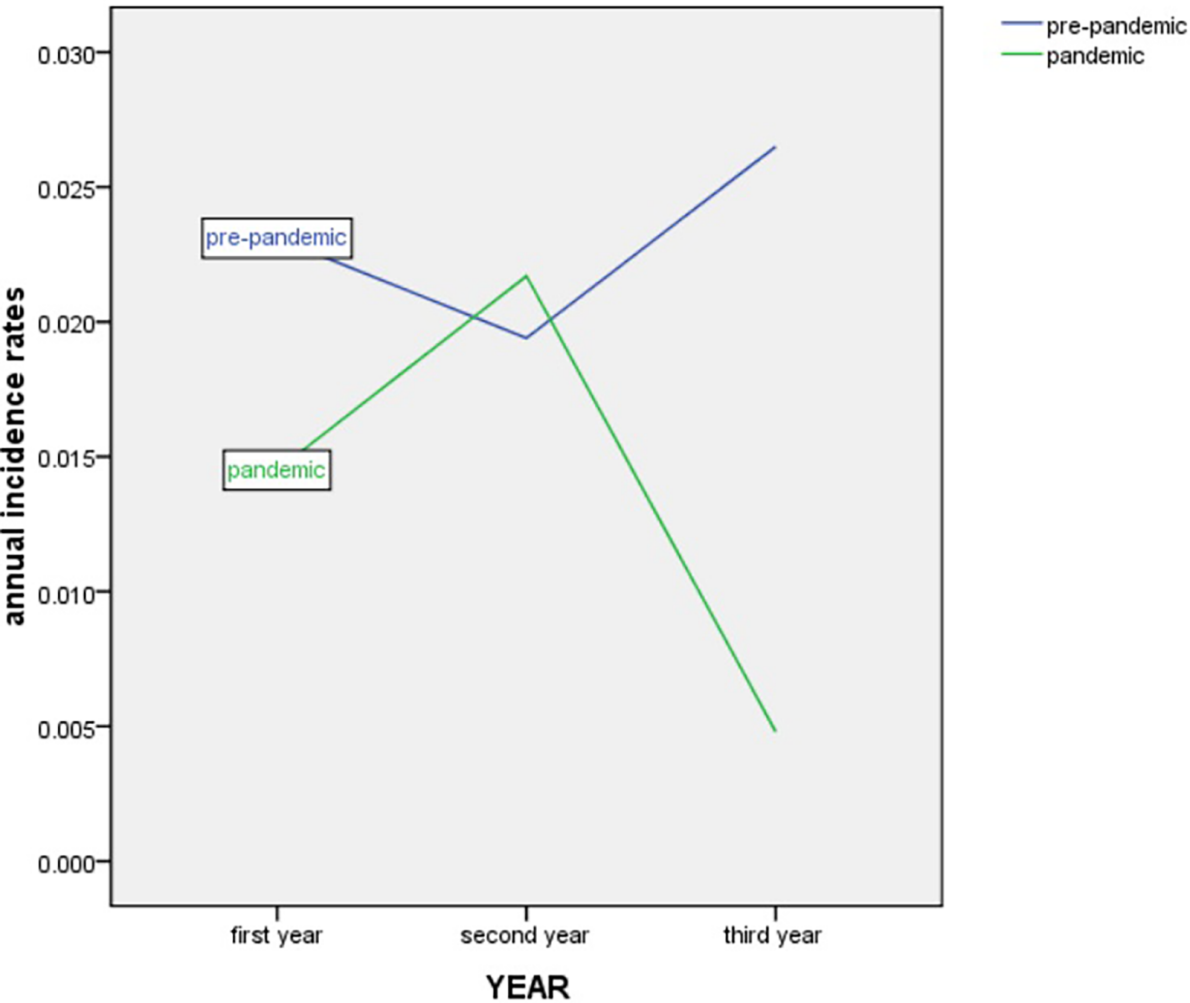
Trend in annual incidence rates of NEC in infants with GA < 37 weeks from March 2017 to December 2022. NEC, necrotizing enterocolitis. GA, gestational age.

The morbidity of premature infants with NEC showed an overall decreasing trend but then increased again in 2021. The annual morbidity of NEC in infants with GA < 37 weeks was 2.31% [45/1,941 (2017)], 1.94% [37/1,899 (2018)], 2.65% [41/1,542 (2019)], 1.45% [23/1,588 (2020)], 2.17% [37/1,702 (2021)] and 0.48% [6/1,231 (2022)] across the 6 years, respectively (Figure 2). Peaks were observed in 2019 and 2021. A trough was observed in 2022 (Figure 3).

### Distribution of pathogens in the two groups

For the pathogens causing NEC, 52 infants with NEC (52/189, 27.5%) had bloodstream infections, of whom 34 (34/123, 27.64%) were born in the prepandemic period and 18 (18/66, 27.27%) were born in the pandemic period. The pathogens in two infants with coinfections in the prepandemic period for whom treatment was abandoned due to their critical conditions were hatch Clostridium, *Staphylococcus capitis*, and *Rhodotorula mucilaginosa*. When prepandemic coinfections were excluded, most patients in the prepandemic period had bloodstream infections caused by gram-negative bacteria (27/32, 84.38%), mainly Klebsiella (10/32, 31.25%) and *Escherichia coli* (5/32, 15.63%). In contrast, most cases in the pandemic period were attributable to gram-positive bacteria (10/18, 55.56%), mainly coagulase-negative staphylococci (8/18, 44.44%) (Table 2).

**Table 2:** NEC aetiology before and during the COVID-19 pandemic.

Groups	Prepandemic period	Pandemic period
Total	34	18
Pathogens	*N* constituent ratio (%)	*N* constituent ratio (%)
Gram negative bacteria		
Klebsiel	10 (29.41)	1 (5.56)
*Escherichia coli*	5 (14.71)	3 (16.67)
*Citrobacter freundii*	3 (8.82)	0
*Serratia marcescens*	3 (8.82)	0
*Enterobacter aerogen*	2 (5.88)	3 (16.67)
*Enterobacter cloacae*	2 (5.88)	0
*Clostridium butyricum*	2 (5.88)	1 (5.56)
Gram positive bacteria		
Coagulase-negative staphylococci	3 (8.82)	8 (44.44)
*Enterococcus faecium*	2 (5.88)	1 (5.56)
*Enterococcus faecalis*	0	1 (5.56)
Mixed infections (>1 pathogen)	2 (5.88)	0

Data are expressed as *n* (%). Most patients in the prepandemic period had bloodstream infections caused by gram-negative bacteria, mainly Klebsiella and *Escherichia coli*. Most cases of bloodstream infections in the pandemic period were attributable to gram-positive bacteria, mainly coagulase-negative staphylococci. NEC, necrotizing enterocolitis; COVID, coronavirus disease.

**Table 3:** Drug resistance of main pathogens before and during the COVID-19 pandemic.

Antibacterials	Klebsiella pneumoniae (*n* = 10)		Antibacterials	Staphylococcus aureus (*n* = 6)	
	Drug resistant strains (strains)	Drug resisitance rate (%)		Drug resistant strains (strains)	Drug resisitance rate (%)
Ampicillin	10	100.00	Penicillin	6	100.00
Piperacillin-tazobactam	1	0.10	Benzoxicillin	6	100.00
Ceftazidime	6	0.60	Erythromycin	1	16.67
Cefepime	5	0.50	Clindamycin	1	16.67
Meropenem	0	0.00	Ciprofloxacin	5	83.33
Imipenem	0	0.00	Levofloxacin	4	66.67
Ciprofloxacin	0	0.00	Cefoxitin Screening	0	0.00
Levofloxacin	0	0.00	Vancomycin	0	0.00
Cefoxitin	3	0.30	Gentamicin	1	16.67
Co-trimoxazole	3	0.30	Tetracycline	0	0.00
Gentamicin	1	0.10	Co-trimoxazole	0	0.00
Tetracycline	4	0.40	Linezolid	0	0.00

Data are expressed as *n* (%). COVID, coronavirus disease.

### Antimicrobial susceptibility of main pathogens in the two groups

All 10 *Klebsiella pneumoniae* isolates from blood samples of NEC patients in the prepandemic period were susceptible to meropenem, imipenem, ciprofloxacin, and levofloxacin according to the minimum inhibitory concentrations and the the Clinical and Laboratory Standards Institute criteria, and the isolates were resistant to ampicillin (100%), ceftazidime (60%), cefepime (50%), tetracycline (40%), cefoxitin (30%), cotrimoxazole (30%), gentamicin (10%) and piperacillin/tazobactam (10%). All six *Staphylococcus capitis* strains were susceptible to vancomycin, linezolid, tetracycline, cotrimoxazole, and cefoxitin, and the isolates were resistant to penicillin and benzathine (both 100%), followed by ciprofloxacin (83.33%), levofloxacin (66.67%) and gentamicin, erythromycin and clindamycin (all 16.67%) (Table 3).

## Discussion

The objective of the current study was to observe the potential effects of pandemic-related measures on NEC morbidity in premature infants in the neonatal ward before and during the COVID-19 pandemic to provide insights for the evaluation of and preventative measures for NEC. This study showed (1) a reduction in NEC (stage II or III) morbidity in all premature infants in the pandemic period compared with the prepandemic period; (2) most cases of NEC with bloodstream infection were attributable to gram-negative bacteria in the prepandemic period but to gram-positive bacteria in the pandemic period; and (3) *Staphylococcus capitis,* the main pathogen isolated from blood specimens of NEC in the pandemic period, was 100% susceptible to vancomycin, linezolid, and cefoxitin. Our study is unique in that it has nearly 6 years of data, including the entire 3-year pandemic period and that it included the largest sample of infants with NEC (stage II or III) to date, with 9,903 premature infants.

In the NICU, we have established hospital and national guidelines not only for the peripartum period, but also strict screening and management of all staff entering the NICU. Key policies in our unit include (1) no visitors are allowed in the NICU; (2) feeding is exclusively formula milk; (3) all COVID-19 positive and exposed patients are grouped together in dedicated areas for treatment and observation; (4) all staff are screened with verbal questions about fever and respiratory symptoms and temperature taken upon entering the NICU; (5) surgical masks are mandatory for all staff and personal protective equipment is worn in the dedicated COVID-19 area of the ward.

A 28-year epidemiological study from tertiary NICUs in the USA found periodic peaks almost every 10 years, but the incidence of NEC remained unchanged (16). Indrio F (17) found that pandemic-related NICU hygiene policies did not reduce the occurrence of NEC in very-low-birth-weight infants in four Italian NICUs. However, the results of some studies are inconsistent with the above findings but similar to those of our study (18). In the first two weeks of life, hospital regimens led to a centre-specific, distinct cluster of faecal microbiome formation in the gut microbiome of very-low-birth-weight infants (17). Quality improvement strategies (e.g., hand hygiene, feeding onset, evidence-based “bundles”, providing an infant with their own mother's milk) displayed the potential for the widespread reduction of NEC (19). The reasons for these different conclusions may be the heterogeneity among studies, including study size, study design, different pandemic-related measures, and differences in participant selection, such as birth weight, gestational age and severity of illness.

Environmental factors may influence the development of NEC (20). The factor most likely to explain the decrease in NEC morbidity in our study is compliance with preventative measures to reduce bacterial and viral transmission. The peak in 2021 means that other more intangible factors may affect the epidemiology simultaneously. Breastfeeding has been implicated as a protective factor against NEC. Interestingly, although breastfeeding rates fell sharply during the pandemic, the average annual incidence of NEC showed a declining trend.

The rates of bloodstream infections in patients with NEC did not change in the pre- and post-pandemic EPOCHs, suggesting that the aetiology of NEC is not exclusively due to infection. As this article focuses more on changes in the incidence of NEC, we have not counted changes in the incidence of LOS in the overall preterm birth population. The COVID-19 related implementation of NICU hygiene policies is likely to reduce the occurrence of LOS in high-risk settings (17, 18). Therefore, we suggest that the control of nosocomial infections may be an explanation for the reduction in the incidence of NEC. Another factor that may contribute to the decrease in NEC rates is the discontinuation of milk-based fortifiers during the pandemic. The relationship between fortifier use and NEC and the optimal point at which multicomponent fortifiers should be added to human milk remains controversial (21, 22).

Most of the current research on the pathogens of NEC has focused on gram-negative bacteria such as cytotoxin-producing Klebsiella oxytoca in the gut (23), C. perfringens (24), E. coli strains (25) and so on. Studies on antibiotic susceptibility in relation to NEC are very limited (26). The antibiotic susceptibility of the second most common pathogen (E. coli) in the pre-epidemic period in our study was not the same as elsewhere (25). A total of 62 (54.86%) of 113 premature infants in a Moroccan NICU were colonised with ESBL-KP (27). Klebsiella spp. recovered from the faeces of preterm infants are genomically more diverse than previously recognised. All faecal K. pneumoniae, K. quasipneumoniae, K. grimontii and K. michiganensis isolates tested were able to colonise and persist in macrophages, indicating their ability to evade the immune system and their potential to cause infection in vulnerable infants. Isolates recovered from infants with NEC and those with no signs of clinical infection encoded multiple beta-lactamases, which may prove problematic when defining treatment regimens for NEC (28). The use of pandemic measures has resulted in a major shift of NEC pathogens towards gram-positive bacteria, which undoubtedly has significant implications for disease management and antibiotic stewardship.

Some limitations should be considered when interpreting our findings. First, the use of Bell's criteria based on clinical and radiographic evidence does have limitations. More recently, abdominal ultrasound has been used in the diagnosis of NEC. However, there are still questions about the sensitivity and specificity of this technique, based on the fact that it needs to be evaluated against a “gold standard”, which we probably do not have, except for intestinal necrosis seen at surgery or autopsy. To address these issues, we selected stage II and III NEC and followed some of these patients to exclude confounding cases such as spontaneous bowel perforation, food protein intolerance enterocolitis syndrome, congenital intestinal anomalies mimicking NEC, etc. Second, currently, microorganisms are best identified by 16S rRNA and 18S rRNA gene sequencing (29). Sequencing can identify fastidious and uncultivable microorganisms, but requires trained laboratory staff and powerful interpretation software, which is expensive and not used in routine clinical practice in NICUs. Third, to fully explore the impact of pandemic-related interventions on NEC morbidity, it is crucial to explore interventions for NEC based on longitudinal studies. Fourth, due to the study design, we cannot detect which factor (visitor restrictions, environmental disinfection, hand hygiene, the use of personal protective equipment, or discontinuation of breastmilk fortification) drives which result, as more than one of these factors changed between the two groups.

## Conclusions

Pandemic-related measures may play a role in decreasing the incidence of NEC in preterm infants and changing the pathogen spectrum. The aetiology of NEC is not limited to environmental factors. However, our study highlights the need for robust multicentre epidemiological investigations of the geographical and cyclical patterns of NEC morbidity so that we may unveil important clues to the aetiopathogenesis of this disease. It is not clear which specific intervention(s) could have contributed to the reduction in NEC, and future studies, such as RCTs, should explore this issue and find methods to sustain the reduction.

## Data Availability

The raw data supporting the conclusions of this article will be made available by the authors, without undue reservation.
